# Feasibility and Inter-Rater Reliability of Physical Performance Measures in Acutely Admitted Older Medical Patients

**DOI:** 10.1371/journal.pone.0118248

**Published:** 2015-02-23

**Authors:** Ann Christine Bodilsen, Helle Gybel Juul-Larsen, Janne Petersen, Nina Beyer, Ove Andersen, Thomas Bandholm

**Affiliations:** 1 Optimized Senior Patient Program, Clinical Research Centre, Copenhagen University Hospital, Hvidovre, Denmark; 2 Physical Medicine & Rehabilitation Research-Copenhagen; Department of Physiotherapy, Copenhagen University Hospital, Hvidovre, Denmark; 3 Musculoskeletal Rehabilitation Research Unit, Department of Physical Therapy & Institute of Sports Medicine, Bispebjerg & Frederiksberg Hospitals, University of Copenhagen, Denmark; 4 Emergency Department, Copenhagen University Hospital, Hvidovre, Denmark; 5 Department of Orthopaedic Surgery, Copenhagen University Hospital, Hvidovre, Denmark; University of Glasgow, UNITED KINGDOM

## Abstract

**Objective:**

Physical performance measures can be used to predict functional decline and increased dependency in older persons. However, few studies have assessed the feasibility or reliability of such measures in hospitalized older patients. Here we assessed the feasibility and inter-rater reliability of four simple measures of physical performance in acutely admitted older medical patients.

**Design:**

During the first 24 hours of hospitalization, the following were assessed twice by different raters in 52 (≥ 65 years) patients admitted for acute medical illness: isometric hand grip strength, 4-meter gait speed, 30-s chair stand and Cumulated Ambulation Score. Relative reliability was expressed as weighted kappa for the Cumulated Ambulation Score or as intra-class correlation coefficient (ICC_1,1_) and lower limit of the 95%-confidence interval (LL_95%_) for grip strength, gait speed, and 30-s chair stand. Absolute reliability was expressed as the standard error of measurement and the smallest real difference as a percentage of their respective means (SEM% and SRD%).

**Results:**

The primary reasons for admission of the 52 included patients were infectious disease and cardiovascular illness. The mean± SD age was 78±8.3 years, and 73.1% were women. All patients performed grip strength and Cumulated Ambulation Score testing, 81% performed the gait speed test, and 54% completed the 30-s chair stand test (46% were unable to rise without using the armrests). No systematic bias was found between first and second tests or between raters. The weighted kappa for the Cumulated Ambulation Score was 0.76 (0.60–0.92). The ICC1,1 values were as follows: grip strength, 0.95 (LL_95%_ 0.92); gait speed, 0.92 (LL_95%_ 0.73), and 30-s chair stand, 0.82 (LL_95%_ 0.67). The SEM% values for grip strength, gait speed, and 30-s chair stand were 8%, 7%, and 18%, and the SRD_95%_ values were 22%, 17%, and 49%.

**Conclusion:**

In acutely admitted older medical patients, grip strength, gait speed, and the Cumulated Ambulation Score measurements were feasible and showed high inter-rater reliability when administered by different raters. The feasibility and inter-rater reliability of the 30-s chair stand were moderate, complicating the use of the 30-s chair stand in acutely admitted older medical patients. However, the predefined modified version of the chair stand test was both feasible and with high inter-rater reliability in this population.

## Introduction

Acute hospitalization of older adults with medical illness is associated with several adverse outcomes, including functional decline, institutionalization, and increased mortality [1–5]. Older medical patients discharged with functional disabilities have a poor prognosis, and up to one-third of the patients will fail to recover to their preadmission level after discharge [1,2]. This indicates a need for early identification of patients with low physical reserve capacity who are at risk of losing function and independence.

In order to identify older medical patients at risk of losing their ability to perform everyday functions and thus their independence, health care professionals need tests that are both feasible and reliable. ‘Feasible’ implies that the tests need to be suitable and tolerable for older acutely admitted medical patients that present with a diverse range of medical conditions and functional levels. In both research and clinical settings, acceptable reliability is a prerequisite for a valid test [6], as consistent test results are required to accurately evaluate patient needs and the effects of treatment.

In the present study, the feasibility and reliability of four frequently used physical performance measures were evaluated during admission to an emergency department. The four measures were isometric handgrip strength (HGS), 4-m gait speed (GS), 30-s chair stand (CS), and the Cumulated Ambulation Score (CAS). Although the Short Physical Performance Battery is often used as a marker for subsequent disability in older community-living people, we used only the 4-m GS test from the Short Physical Performance Battery [7–10]. Notably, testing only GS performs almost as well as the full Short Physical Performance Battery in predicting subsequent disability [10,11]. The four measures were chosen based on their ability to evaluate and predict adverse health outcomes, including increased dependency in mobility and function [7,10,12–15], in both hospitalized older patients [16–18] and in community-living older people [4,10,12,15].

We assumed that these four measures were applicable to older patients with a diverse range of medical illnesses and functional levels, including those who are bedridden. In addition, the measures are assumed to be fast and easy to administer for health-care professionals, and the space and equipment requirements are very modest. However, there are few studies of the feasibility of these measures for assessing acutely hospitalized older patients [16–19], and the reliability of the investigated measures have typically been based on data from studies conducted in community-living older people with stable health [16,19]. In contrast, the status of an acutely admitted patient often changes during hospitalization, which may influence the feasibility and reliability of the measurements and, thus, the evaluative and predictive values in this population. Indeed, reports of critically ill patients admitted to intensive care units show high measurement errors in grip strength assessment compared with errors reported in community-living older adults [20,21]. Consequently, it is important to establish the feasibility and reliability of these measures in a population of acutely admitted older adults before investigating the predictive value of physical performance measures in this population. Accordingly, the aim of this study was to determine the feasibility and inter-rater reliability of four simple measures of physical performance in acutely admitted older medical patients.

## Methods

### Ethics statement

This study complies with the ethical rules for human experimentation stated in the Declaration of Helsinki. Signed informed consent was obtained from all participants prior to inclusion. The Danish Data Protection Agency and the Research Ethics Committees for The Capital Region approved the study (registry number H-1-2011-167).

### Setting and participants

This study was performed in a 30-bed emergency department at Hvidovre Hospital, University of Copenhagen, Denmark from March 2012 to April 2012. The majority of acutely hospitalized older medical patients (65+ years) are admitted through the emergency department. There are approximately 4000 such admittances annually, and up to 50% of all acutely admitted older medical patients (65+ years) are discharged the day after their admission.

The present study was planned as a preliminary feasibility and reliability study to analyze the viability of the measurement methods for a larger prospective cohort study that will look at the prediction of adverse health outcomes in relation to acute medical admission.

This study included patients using random sampling. All were aged ≥65 years and were admitted for an acute medical illness. The exclusion criteria were an inability to cooperate, expected hospitalization of less than 24 hours, an inability to understand Danish, admission to an intensive care unit, a cancer diagnosis, or terminal disease. Eligible patients were asked if they were interested in participating, and informed consent was obtained from eligible patients who wished to participate. More patients met the inclusion criteria than could be included with respect to rater resources; accordingly, patients were randomly selected based on their social security number by the use of a computer-generated list.

The required sample size was estimated by calculating the expected width of the 95% confidence intervals of the ICCs from the repeated measurements of HGS, GS, and 30-s CS [22]. With expected ICCs of 0.85, a minimum acceptable ICC level of 0.7, two replicates per measure, and a maximum width of the 95% confidence intervals of 0.3, a sample of 40 patients was needed [22]. To account for dropout, 52 patients were included. If the required sample size of 40 patients was not met for one of the measures, the test was considered not feasible in this population.

### Design

**Fig 1:**
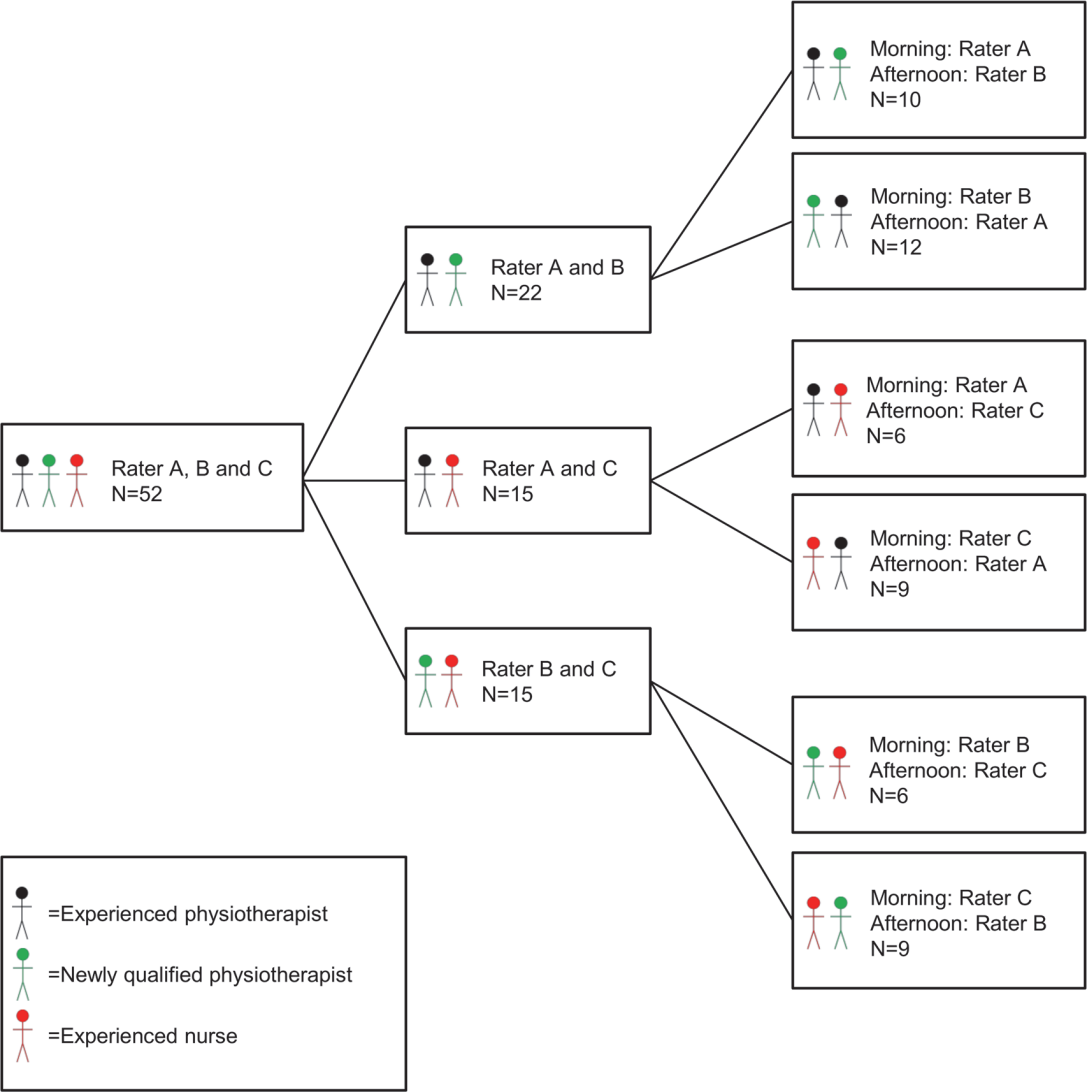
Allocation of raters.

The study used an inter-rater intra-day design in which all patients were tested at two sessions that were 2–3 hours apart (e.g. at a morning session and again at an afternoon session) during the first 24 hours after hospital admission. To reflect the range of health professionals working in the emergency department and to model the reality of potential future implementation, three different raters were trained to assess the patients: an experienced physiotherapist (A), a newly qualified physiotherapist (B), and an experienced nurse (C); these formed three pairs of raters (Fig. 1). Each patient was tested in the emergency department at the ward of observation by one pair of raters, and the individuals that made up each pair of raters were blinded to each others results. The raters that performed the morning and afternoon assessments were decided randomly by drawing lots. All raters received extensive training in the testing protocols and were calibrated before the start of the study. The reporting of the study follows the Guidelines for Reporting Reliability and Agreement Studies [23].

### Physical performance measures

HGS was assessed in the dominant hand using a handheld dynamometer (Saehan, Digi-II). The HGS is a reliable measure in community-living older adults [21]. Patients who were able to leave the bed sat in a chair with their elbow flexed at 90°. The lower arm was placed on the armrest with the wrist in a neutral position. The contralateral hand was placed in the lap with the palm facing upwards. Bedridden patients were assessed in a sitting position with the backrest elevated. The position of the backrest and the elbow angle were measured. If needed, the dynamometer was supported manually. Patients were instructed to squeeze the handle as forcefully as possible for 5 seconds. After one practice trial, three valid trials were recorded. If the third trial elicited the highest value, additional trials were performed. The highest value in kilograms was used as the data point.

**GS**, using a walking aid if one was normally used, was assessed over a 4-m course. The GS is a reliable measure in community-living older adults [24]. Patients were asked to stand with their feet behind a starting line and to start walking at their usual pace at the raters command, “3-2-1-go”. To reduce the effect of deceleration, each patient was instructed to walk towards a visual goal and was stopped after walking 5.5 m. The timer was started upon the first foot-step and stopped when the first foot crossed the 4-m end line. The faster of two trials was used as the data point.

**The 30-s CS** was assessed using a standard chair (height of 45 cm) [25]. The CS is reliable in older persons with chronic conditions and is a valid surrogate measure of lower extremity strength in healthy and chronically ill older persons [25,26]. The predictive ability of the CS test in community-living older persons has been evaluated based on the timed test of five repetitions of chair stands [7]. However, the test is not useful for assessing frail older patients who can only perform one to four repetitions. Therefore, we used the 30-s CS test. The patient was encouraged to stand up from a chair as many times as possible without stopping, keeping the arms folded across the chest. After careful instructions, the patient got one test trial to evaluate their ability to follow the test protocol. The timer was started when the rater said “3-2-1-start,” and the patient was stopped after 30 sec. Only full standing positions were counted. The analysis used the number of repetitions performed with the arms folded across the chest. To evaluate data from patients who were unable to follow the protocol i.e. to complete the initial test trial, we used three categories: (1) an ability to rise without using the armrest with arms folded across the chest; (2) an ability to rise using the armrest; and (3) an inability to rise independently from the chair. This categorization is referred to as CS_G_.

**Basic mobility** was evaluated using the CAS. The CAS is a reliable [27] and valid predictor of length of hospitalization, time-to-discharge status, 30-day mortality, and postoperative medical complications in older patients with hip fracture [18]. The CAS quantifies the patient’s independence in performing three activities: getting into and out of bed, sit-to-stand from a chair, and walking. Each activity is scored on an ordinal scale from 0 to 2 (0 = not able to, 1 = with guidance/support, 2 = independently), and the resulting total score ranges between 0 (no basic mobility) and 6 (independent basic mobility). The CAS was scored after the patient had returned to bed using observations during the prior physical performance tests.

### Vital signs and pain

The Early Warning Score (EWS) is a systematic observation and risk assessment used in the emergency department to monitor deterioration during acute illness and to assess mortality risk [28]. The EWS is based on seven vital signs: level of consciousness, temperature, heart rate, arterial blood pressure, respiratory rate, peripheral oxygen saturation, and oxygen therapy [28,29]. The EWS was assessed prior to the physical performance measurements at both the morning and afternoon sessions and was used to evaluate deterioration between the two sessions. The EWS is scored on an ordinal scale from 0 to 20, with higher scores reflecting more severe illness and higher mortality risk. At both the morning and the afternoon sessions, before the four physical performance tests, the patients were asked if they were in pain and, if so, they were asked about the localization of the pain. The pain level was scored on a five-point Verbal Ranking Scale, where 0 = no pain, 1 = light pain, 2 = moderate pain, 3 = severe pain, and 4 = intolerable pain [30].

### Descriptive data

During the morning session, a structured interview was used to collect information about cognitive status and functional independence. Cognitive status was assessed with the Short Orientation-Memory-Concentration Test [31]. Functional independence relating to indoor and outdoor mobility was assessed with the New Mobility Score (NMS) to determine the patients’ premorbid functional independence (based on the patient’s recall of the two weeks before the current admission) and functional independence at admission [32,33]. The interview was followed by assessments of vital signs and by the four physical performance measures. The measures were administered in a pre-defined order to meet the needs of bedridden patients and to reduce fatigue. The order was HGS, GS, CS, and CAS. A short rest of up to five minutes was allowed between each test.

### Statistics

Data are presented as means with standard deviations for continuous data and as medians with interquartile ranges for ordinal data or for data that were not normally distributed. Dropouts and non-dropouts were compared with respect to categorical variables using the Chi-squared test or Fischer’s exact test. The Kruskal-Wallis test was used for ordinal and ratio-interval variables if the data were not normally distributed.

In this study, feasibility was assessed based on an evaluation of pain and discomfort during testing and on the patient’s ability to perform the individual measurements. Feasibility was categorized as follows based on the percentage of patients who were able to perform the test: very low (<25%), low (≥25% but ≤50%), moderate (≥50% but <75%), or good (≥75%). An overall increase in pain during the assessment that prevented further testing was considered unacceptable. A mixed effect model (with time as the fixed effect and patients as the random effect) was used to estimate the difference in pain within the morning and the afternoon sessions. A paired t-test was used to test the difference in pain between the morning and afternoon sessions.

A mixed effect model (with raters and time of day as fixed effects and patients as the random effect) was used to estimate the difference between raters and to assess systematic bias between the morning and afternoon tests for continuous data. A proportional odds model was used for ordinal variables. Bland-Altman plots were used to assess the heteroscedasticity of the data. Relative reliability was expressed using kappa statistics for ordinal variables. The weighted kappa for CAS and CS_G_ was calculated with the corresponding 95% confidence interval (95% CI) and presentation of absolute agreement. For ratio-interval data, GS, CS, and HGS relative reliability was assessed using the intraclass correlation coefficient (ICC 1.1) with the corresponding 95% CI. For this purpose, a random effect model was used with the participant and the rater as random effects variables. Model 1.1 was used because all raters did not assess each patient, and the ICC was calculated based on the trial with the best performance for each test. The absolute reliability was expressed as the standard error of measurement (SEM) and was calculated by SEM = SD ×√(1-ICC), where SD is the standard deviation of calculated using all test scores from both the morning and afternoon sessions. SEM was used to calculate the smallest real difference (SRD_95_), using the equation SRD_95_ = SEM × √2 × 1.96. The SRD is an estimates estimation of the smallest real difference required to be 95% confident that an observed change in an individual score reflects a real change in the underlying parameter. SEM and SRD are presented as percentages of their respective means (SEM% and SRD%); these were calculated, dividing the SEM and SRD by the mean of all test scores from both testing sessions and multiplying by 100. ICCs that were at least 0.7 and an SEM% that was 10% at most were considered acceptable.

The influence of the acute illness or condition on the re-test differences for each of the four test scores was evaluated using a logistic regression model for CAS and CS and a generalized linear regression model for HGS and GS. The EWS score from the morning session was used as the explanatory variable, and the response variables were the numeric differences between the morning and the afternoon sessions for the HGS, GS, CS, and CAS.

The level of significance was set at 0.05, and all statistical tests were two-tailed. The statistical analyses were performed using SAS 9.3 for Windows.

## Results

### Attendance and drop-out

**Fig 2:**
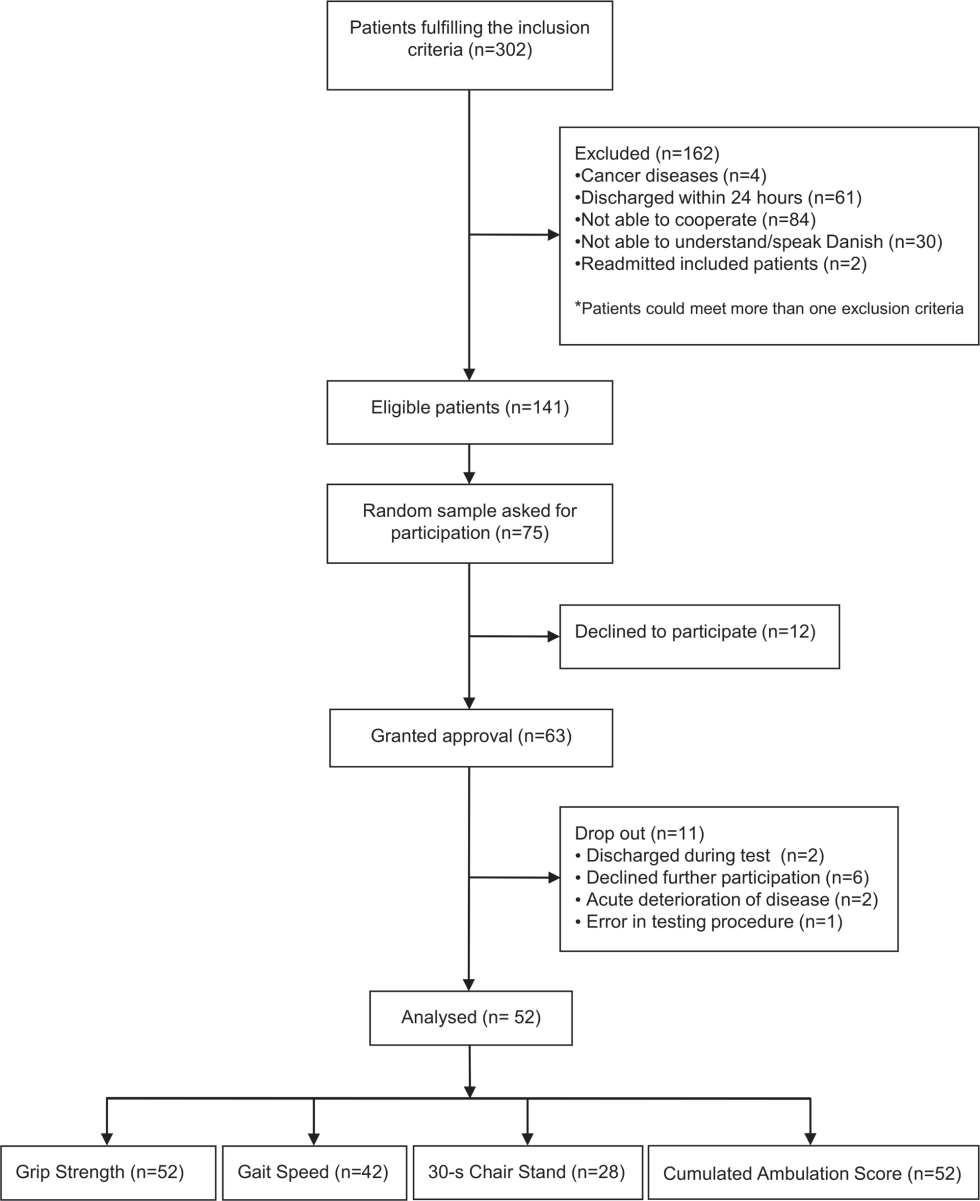
Inclusion of patients in the study (N = 52).

On inclusion days, 302 medical patients (+65 years) were admitted to the emergency department (Fig. 2). A total of 75 randomly selected patients were asked if they were interested in participating. Of the 75, 63 patients granted approval to participate in the study; of these, 52 patients participated in the testing. The characteristics of the study sample are shown in Table 1. The patients who dropped out before the afternoon session (n = 11) did not differ significantly from the remaining patients regarding age (79 versus 78 years, p = 0.55), sex (women: 55% versus 60%, p = 0.75), cognitive impairment (46% versus 40%, p = 0.75), premorbid NMS (4 versus 6 points, p = 0.36), and NMS at admission (2 versus 3 points, p = 0.26). The reasons for dropping out of the study included: acute deterioration in the patient’s condition that prevented further participation (n = 2), decline of further participation (n = 6), discharge (n = 2), and error in the testing procedure (n = 1).

**Table 1:** Characteristics of the study population (N = 52).

	N (%)	Value
**Age**, years; mean (SD)		78.0 (8.3)
**Female**; number (%)		38.0 (73.1)
**BMI**, kg/m^2^; mean (SD)		25.1 (4.7)
**NMS**, 14 days before admission, points; median (IQR)		6 (3;9)
**NMS at admission**, points; median (IQR)		3 (2;8)
**Use of walking aid**, number (%)		25 (48.1)
**OMC score**, points < 18; number (%)		16 (30.8)
**Reason for admission**, number (%):		
Cardiovascular		12 (23.2)
Infection (pneumonia, erysipelas, acute cystitis)		23 (44.2)
COPD		6 (11.5)
Other (diabetes, musculoskeletal, gastrointestinal, falls, dehydrated, anemia)		11 (21.1)
**Isometric handgrip strength, HGS**		
Patients able to perform HGS test, kilograms; mean (SD)	52 (100)	21.58 (8.12)
**Gait speed, GS**		
Patients able to perform GS test, m/s; median (IQR)	42 (81)	0.65 (0.51;0.81)
**Chair stand, CS**		
Patients able to perform the CS test, repetitions; mean (SD)	28 (54)	7.41 (3.10)
**Cumulated Ambulation Score, CAS**		
Patients able to perform CAS, number, points; median (IQR)	52 (100)	6 (4;6)

Abbreviations: IQR: interquartile range. SD: standard deviation. OMC: Orientation-Memory-Concentration-test. NMS: New Mobility Score. BMI: body mass index. COPD: chronic obstructive pulmonary disease.

### Feasibility

Table 1 shows the number of patients who completed each of the four tests. All patients completed the HGS assessment; 6 patients were tested while in bed. GS was assessed in 42 patients, 40 of whom participated in a repeated test. Twelve patients did not perform the repeated GS testing; Seven were bedridden, 3 were unable to walk, 1 patient declined participation, and 1 patient could not cooperate with the test instructions during the morning session. CS was assessed in 45 patients; 28 were able to follow the protocol and performed the test with arms folded across the chest; 15 needed the armrest to stand up from the chair; and 2 needed assistance to stand up from the chair. Seven patients were bedridden and unable to participate. CAS was assessed in 52 patients; of these, the result of the morning test was missing for 1 patient because of rater error. There was no change in patient-reported pain within the morning session (p = 0.93) and within the afternoon session (p = 0.73). The patient-reported pain did not change significantly between the morning and afternoon sessions (p = 0.17).

### Deterioration in the acute illness

The total EWS score from the morning session had no significant influence on the numeric retest differences for any of the four measurements: HGS: 1-exp *β*: 5%, (95%CI: −6%-17%) p = 0.35; GS: 1-exp *β*: 7% (95% CI: -10%-27%), p = 0.45; CS: OR 0.61, (95% CI: 0.31–1.19), p = 0.14; CAS: OR 1.13, (95% CI: 0.82–1.55), p = 0.45. The vital signs did not change between the morning and afternoon sessions (p >0.22).

### Inter-rater reliability

Heteroscedasticity was present for GS; accordingly, all further analyses of GS were performed using log-transformed data. No systematic bias was found between the morning and the afternoon sessions for any of the four measurements of physical performance (HGS, p = 0.28; GS, p = 0.42; CS, p = 0.20; and CAS, p = 0.50; Table 2). The relative and absolute reliability are shown in Table 3. The relative inter-rater reliability for HGS and GS were 0.95 and 0.91, respectively, with an absolute inter-rater reliability (SEM%) of 8% and 7%, respectively. The relative and absolute inter-rater reliability for CS were based on 28 patients, as 17 patients had missing values because they were unable to follow the protocol. The ICC_1–1_ was 0.82, with an SEM% of 18% and an SRD% of 49%. Data from 45 patients were used in the CS_G_. The observed agreement for CS_G_ was 93%, and the weighted kappa was 0.88 (0.76–1.00). The observed agreement for CAS was 77%, and the weighted kappa was 0.76 (0.60–0.92).

**Table 2:** Feasibility and relative and absolute inter-tester reliability for the measurements of physical performance.

	Feasibility N (%)	β	P-value	ICC_1.1_ (LL_95_)	SEM (SEM%)	SRD_95_ (SRD%)	Weighted kappa (95% CI)	Observed agreement (%)
HGS	52 (100)	0.39	0.28	0.95 (0.92)	1.74 (8)	4.83 (22)	-	-
CS	28 (54)	-0.42	0.20	0.82 (0.67)	1.32 (18)	3.67 (49)	-	-
GS	42 (81)	1.02	0.42	0.91 (0.73)	^a^(7)	^a^(17)	-	-
CAS	52 (100)	0.16	0.50	-	-	-	^b^0.76 (0.60–0.92)	77
CS_G_	45 (87)	-	-	-	-	-	0.88 (0.73–1.00)	93

HGS: isometric handgrip strength (kg), CS: 30-s chair stand test (repetitions), GS: gait speed (Exp β), CAS: Cumulated Ambulation Score (points), CS_G_: Chair stand test, categorized as the ability to do the following: rise without using the armrest with arms folded across the chest, rise using the armrest, or inability to rise independently from the chair, β: mean differences between morning and afternoon session using a mixed effect model, ICC_1.1_: intraclass correlation coefficient model 1.1, LL_95_: Lover 95% confidence limit, SEM: standard error of measurement, SEM%: standard error of measurement expressed as a percentage of the mean, SRD_95_: Smallest real difference at the 95% confidence level, SRD%: smallest real difference as a percentage of the means.

^a^SEM and SRD were not calculated as GS is log-transformed.

^b^The calculation of the weighted kappa was based on N = 51, as 51 patients participated in the repeated testing

**Table 3:** The mean differences between raters as determined using a mixed effect model.

	HGS (n = 52)	GS (n = 42)	CS (n = 28)	CAS (n = 52)
**Rater A**	Reference	Reference	Reference	Reference
**Rater B**	-0.18	1.00	-0.12	0.21
**Rater C**	-0.63	1.01	-0.43	-0.05
**P-value (main effect)**	0.46	0.99	0.66	0.67

All test values are β coefficients and express the mean differences between the reference rater (A) and rater B or rater C. HGS: isometric handgrip strength (kg), GS: gait speed (exp β), CS: 30-s chair stand test (repetitions), CAS: Cumulated Ambulation Score (points).

### Mean differences between raters

Fig. 1 shows the number of patients tested by each rater and by each rater pair. The mean differences between the three raters based on the tests and retests of the four physical performance measurements are shown in Table 3. No systematic differences between the three raters were found for any of the measurements (β values); HGS (kg): Rater A (reference) = 0, rater B = -0.18, and rater C = -0.63 (p = 0.46); GS (exp β): Rater A (reference) = 1, rater B = 1.01, and rater C = 0.99 (p = 0.99); CS (repetitions): Rater A (reference) = 0, rater B = -0.12, and rater C = -0.43 (p = 0.66); and CAS (points) Rater A (reference) = 0, rater B = 0.21, and rater C = -0.05 (p = 0.67) (Table 3).

## Discussion

This study investigated the feasibility and inter-rater reliability of clinical measures of physical performance as assessed by different healthcare professionals. There were two primary findings. First, the HGS, GS, and CAS measurements were feasible and had substantial inter-rater reliability in acutely admitted older medical patients. Second, the feasibility and inter-rater reliability of the CS test was moderate, as only about half of the patients in this population could perform the test; in addition, there were large measurement errors.

### Isometric grip strength

Acutely admitted older medical patients are a heterogeneous population with a wide range of physical abilities who show large variations in terms of the impact of the acute medical illness on their physical performance. Despite this, the HGS had an ICC level similar to that reported for community-dwelling older adults(who were retested after one week) and similar to results from an intra-day inter-rater study of critically ill patients (ICC 0.94 and 0.92 respectively)[20,21]. However, a high ICC could be the result of sample heterogeneity [6,34]. Therefore, evaluation of the absolute reliability is important to allow for comparisons between populations. The SEM in the present study was similar to that reported previously in community-living older adults (1.74 kg versus 1.89 kg) [21], but it was substantially lower than the SEM% found in critically ill patients (SEM%, 8% versus 21%) [20]. These findings support the use of HGS as a feasible and stable measure that is not noticeably affected by acute medical illness.

### Gait speed

GS is a commonly used measure for evaluating older adults, both in clinical practice and in research settings, and GS is interpreted as a surrogate indicator of health [6,8,18]. In this study, a floor effect was found for GS. The patients who were unable to perform the GS test had severe medical symptoms and were wheelchair users. This was an expected finding in our study population, which has a diverse range of medical conditions and functional levels. Our results support the conclusion that GS is feasible for use in the majority of older medical patients. The relative reliability of the GS was high and comparable to that reported in an intra-day test-retest study performed in community living older adults (ICC 0.87) [24]. With respect to the absolute reliability of the GS, heteroscedasticity was observed, indicating that measurement error increases when gait speed decreases. This implies that the absolute reliability should be interpreted with caution, especially in older medical patients, who walk slowly. For the GS test, the SEM% values indicate that we can expect a variation of 7% of the mean at the group level and a variation of 17% of the mean at the individual level before the measurement error has been exceeded. There is no consensus on what constitutes acceptable SEM% and SRD% values, but in healthy community living older adults, SEM% and SRD_95%_ values of 4% and 13%, respectively, have been reported for homoscedastic data [24,6], suggesting that greater variation between scores in older patients with medical illness may be expected. If measures of physical performance must be used to identify at-risk patients with a low level of function, an ICC of a minimum of 0.7 is required [35], whereas for evaluative purposes, the degree of required reliability depends on the size of the absolute measurement errors and the clinically relevant changes. With respect to the latter, clinically relevant changes for GS of 0.08 m/s and 0.26 m/s have been reported in sedentary community living older people and in older women undergoing rehabilitation after hip fracture, respectively [36–38]. This indicates that GS cannot be used to evaluate changes in people who are slow walkers. However, clinically relevant changes in GS for the population of acutely admitted older medical patients remain unknown.

### Cumulated Ambulation Score

During hospitalization, special attention should be paid to patient independence in basic mobility, as this is an important clinical sign of the need for assistance in performing everyday mobility tasks both during admission and after discharge. The results of the present study showed that CAS was feasible and confirm the findings reported in geriatric patients and older patients with hip fracture [27,39]. The CAS also showed substantial relative reliability, though the estimate was below that of a similar aged hospitalized population (0.77 versus 0.95) [27]. The present study used a repeated design in which each measure was administered twice by different raters, unlike the study by Kristensen et al. [27], where the same trial was evaluated by two different raters. Thus, increased reliability is to be expected when between-trial variation is reduced.

### Chair Stand

As in the present study, another study also found low feasibility for the CS test in geriatric patients, only 19% of whom could perform the CS [39]. The absolute reliability was lower than in studies of medically stable older adults [40,41], suggesting that the CS is less reliable in older medical patients. In older people with osteoarthritis, a change in the CS of 2–2.6 repetitions was reported to be clinically relevant [42]. The present study found that an individual patient needs to change by 3.7 repetitions to exceed the SRD_95%_. However, the sample size was small because of patients who were not able to follow the protocol; this influences the reliability estimate. The low feasibility suggests that the CS should not be the first choice for measuring physical performance in older medical patients. High reliability was obtained for the CT_G_ where the patient’s ability to rise from a chair was classified into three categories. This is interesting because the inability to rise from a chair was associated with the highest mortality risk in older community living people [43]. Accordingly, this simple evaluation of CS ability may give health professionals a quick indication of risk status and lower-extremity muscle strength [25,26,44]. However, studies are needed to show the usefulness of the CT_G_ in risk evaluation in older medical patients.

### Study limitations

The limitations of the present study included the potential for deterioration in the acute medical condition of the included patients. A 2–3 hour rest interval was chosen to balance the risk of changes in clinical status and also to allow the patients to recover. The vital signs of the patients did not change significantly during repeated testing, and there was no association between the EWS score and the retest differences in any of the four measurements. This indicates that the patients were medically stable. The four measurements of physical performance were administered in a predefined order, which could induce fatigue development during the session. The protocol included a habituation trial for HGS, GS, and CS to reduce potential learning effects, which are frequently observed in reliability studies that include physical performance measurements [6,34]. This may explain why there was no systematic bias in any of the repeated measurements. Our choice to use different types of healthcare professionals with different levels of experience as raters increases the generalizability of our results to similar populations and settings and resembles the busy acute clinical setting in which these measures would likely be used. No systematic mean differences were found between raters; however, the study was not powered for this purpose. The majority of patients were able to perform the measurements, and there was no change in pain level. There were few dropouts, and drop out was not related to the assessments.

## Conclusion

We found good feasibility for the GS and HGS measurements and for the CAS measurement of basic mobility, with substantial inter-rater reliability in acutely admitted older medical patients when administered by different health-care professionals. The feasibility and inter-rater reliability of the 30-s CS were moderate, as only 54% of the patient could perform the test. This complicates the use of the 30-s CS measurement in acutely admitted older medical patients. However, the predefined modified version of the chair stand test was both feasible and with high inter-rater reliability in this population.

## Supporting Information

S1 Dataset Data and formats. (XLSX)

S2 Dataset ICD-10 codes version 2010. (XLS)
